# Use of a Lateral Orbital Flap for Lateral Canthal Reconstruction: A Case Report

**Published:** 2020-05-27

**Authors:** Yuta Nakanishi, Natsuko Kakudo, Kenji Kusumoto

**Affiliations:** Department of Plastic and Reconstructive Surgery, Kansai Medical University, Osaka, Japan

**Keywords:** lateral orbital flap, lateral canthal reconstruction, the facial nerve, flap necrosis, SMAS (superficial musculoaponeurotic system)

## DESCRIPTION

**Figure 1:**
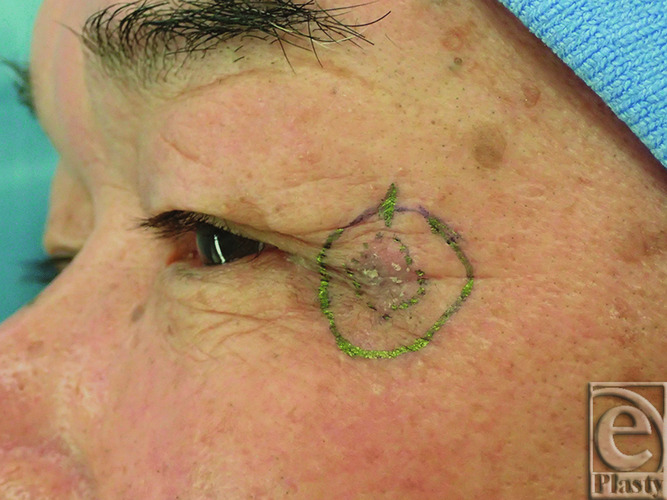
Preoperative squamous cell carcinoma.

**Figure 2:**
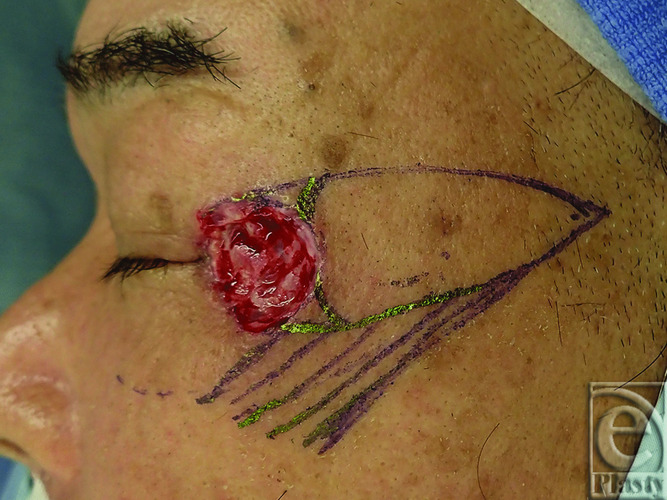
Design of the lateral orbital flap.

**Figure 3:**
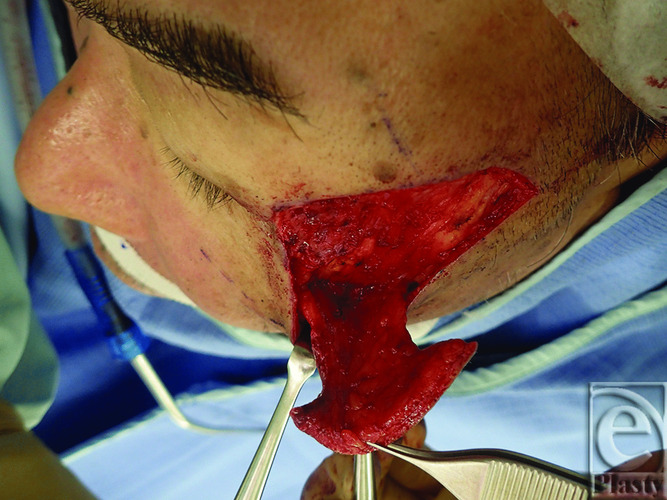
Elevation of the lateral orbital flap.

**Figure 4:**
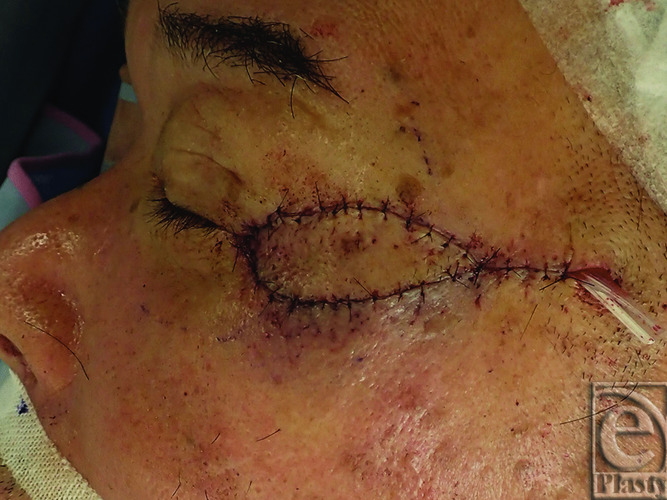
The lateral orbital flap covering the defect.

**Figure 5:**
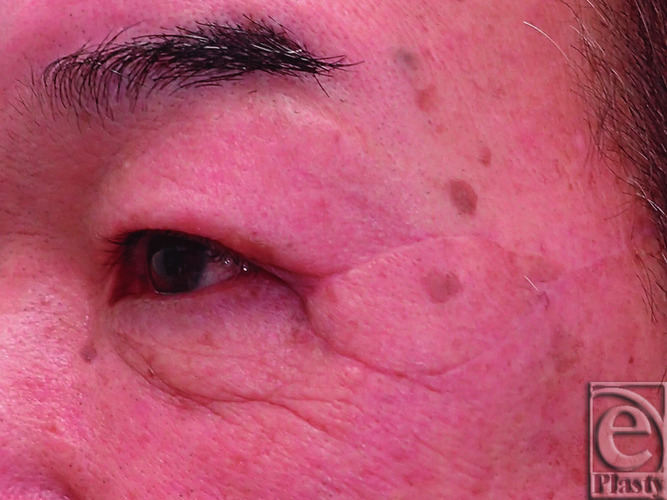
Seven months postoperatively.

A 72-year-old man visited our hospital with a 10-mm erythema lateral to the left lateral canthus a year prior. Biopsy yielded a diagnosis of squamous cell carcinoma (SCC). The tumor was excised with a 5-mm safety margin, and no tumor was intraoperatively confirmed at the wound margins (Fig 1). To reconstruct the excised defect of 2 × 2 cm in size, a lateral orbital flap was created to match the color and texture. The island flap was designed outward from the defect (Fig 2). A subcutaneous pedicle including the subcutaneous fat tissue and part of the orbicularis oculi muscle was prepared. The island part was elevated (Fig 3) and transferred to the defect, the wound was closed, and the drain was placed at the pedicle site (Fig 4). Good color and texture matching was achieved, and condition of the flap 7 months after the operation is shown in Figure 5.

## QUESTIONS

1. How is the lateral orbital flap elevated?
2. What are the complications and how to avoid them during reconstruction with the lateral orbital flap?
3. What methods are available to reconstruct lateral periorbital defects?
4. What are the advantages and applications of lateral orbital flaps?

## DISCUSSION

A lateral orbital flap is a local island flap used for reconstruction of the lateral canthus invented by Ogawa et al^1^ in 2011. The flap is designed between the lateral canthus and the sideburn as a spindle or crescent-shaped island of 2 to 3 cm in width and 5 cm in length.^1^ The shape and size of the flap depend on the defect site. For peri-eyelid reconstruction, a flap as thin as possible is elevated above the SMAS (superficial musculoaponeurotic system) layer. The SMAS in the parotid and cheek areas is always present and is continuous with the posterior part of the frontalis muscle in the upper part of the face and the platysma muscle in the lower part of the face.^2^ For the transfer, pedicles should be elevated and preserved mediocanthal to the flap. The island is transferred to the defect by sliding or rotating, and the donor site is then primarily closed with no conspicuous scar.^1^

Complications of lateral orbital flaps include facial nerve palsy, flap necrosis, and hematoma formation. The temporal branch of the facial nerve runs from the bifurcation through the outer fascia of the parotid and under the temporal parietal fascia on the zygomatic arch and reaches the frontalis muscle. To avoid damaging the facial nerve, the island flap and the subcutaneous pedicle should always be managed above the SMAS. Alternatively, it passes through the level of the SMAS at 3 to 4 cm lateral to the orbit and should therefore be drawn inside. Externally, the flap must include the shallow layer of the subcutaneous tissue.^3^ To prevent flap necrosis, sufficient subcutaneous exfoliation is performed after moving the flap such that the skin and subcutaneous pedicles are not strained. By including the dense vascular network in the flap, the risk of flap necrosis can be minimized.^1^^,^^4^ To prevent hematoma formation, satisfactory hemostasis during surgery should be provided and, if necessary, a drain should be inserted, as in this case.

Reconstruction methods for lateral orbital defects include lateral orbital flaps, bilobed flaps, rotation cheek flaps, rhomboid flaps,^1^ island mucochondrocutaneous flaps,^1^ and semicircular flaps.

Lateral orbital flaps are advantageous in that good color and texture matches can be obtained as a local flap. As the subcutaneous fat tissue around the eyelid has an abundant vascular network,^5^ these flaps are more stable than others, have improved mobility,^4^ and have a reduced risk of necrosis by preserving blood flow in the subcutaneous pedicle. During elevation of the lateral orbital flap, the operator must preserve the blood circulation from the orbicularis oculi muscle, which supports the flap, and elevate it without damaging the facial nerve.^4^ The donor site is directly closed and the scar is not conspicuous. Lateral orbital flaps are useful not only for skin defects after trauma and tumor removal but also for defects in the upper and lower eyelids, including the socket base and the lateral canthus.^4^ With careful attention to complications, lateral orbital flaps should be considered for repairing peri-eyelid defects.

## SUMMARY

Lateral orbital flaps are a good reconstruction method for repairing defects of the lateral canthus area because of the good color and texture matches and thickness.
